# Associations of Early-Life Risk Factors with Racial Disparities in Incident Dementia among Older Americans

**DOI:** 10.1093/geroni/igaf122.1319

**Published:** 2025-12-31

**Authors:** Yi Wang, Yuting Qian, Zhuoer Lin, Xi Chen

**Affiliations:** Yale University, New Haven, Connecticut, United States; Yale School of Public Health, New Haven, Connecticut, United States; University of Illinois Chicago, Chicago, Illinois, United States; Yale University, New Haven, Connecticut, United States

## Abstract

Early-life risk factors, such as exposure to trauma or receiving less and poor-quality education, have been linked to brain development and academic achievement, both of which may influence dementia risk later in life. However, there remains a limited understanding of the associations between these early-life risk factors and incident dementia, as well as their role in driving racial disparities in dementia incidence. This study aimed to examine the associations between early-life risk factors and incident dementia, and to assess how these factors mediate racial disparities in dementia incidence. Using data from the 2000-2020 Health and Retirement Study (HRS), we analyzed a sample of 5,578 participants aged 70 years and older, excluding those with dementia at baseline. We determined dementia status using previously validated algorithms (the Expert Model) designed for racial/ethnic disparities research in HRS. Our survival analyses suggest that the age- and sex-adjusted hazard ratio (HR) for incident dementia was 2.12 (95% CI, 1.76-2.55) for Black compared to White individuals. Mediation analyses showed that attending any segregated schools before college explained 42.2% of this racial disparity, while being born in the US South and having less than a high school education accounted for 36.8% and 21.9% of the disparity, respectively. These findings emphasize the crucial role of early-life factors in the screening, prevention, and intervention of dementia onset, and suggest that educational experiences and early-life environments are key mechanisms contributing to racial disparities in dementia incidence.

